# Effect of Immune Checkpoint Blockade on Myeloid-Derived Suppressor Cell Populations in Patients With Melanoma

**DOI:** 10.3389/fimmu.2021.740890

**Published:** 2021-10-12

**Authors:** Steven H. Sun, Brooke Benner, Himanshu Savardekar, Gabriella Lapurga, Logan Good, David Abood, Erin Nagle, Megan Duggan, Andrew Stiff, Mallory J. DiVincenzo, Lorena P. Suarez-Kelly, Amanda Campbell, Lianbo Yu, Robert Wesolowski, Harrison Howard, Hiral Shah, Kari Kendra, William E. Carson

**Affiliations:** ^1^Department of Surgery, Division of Surgical Oncology, The Ohio State University, Columbus, OH, United States; ^2^Comprehensive Cancer Center, The Ohio State University, Columbus, OH, United States; ^3^Center for Biostatistics, Ohio State University Wexner Medical Center, Columbus, OH, United States

**Keywords:** nivolumab, pembrolizumab, immune checkpoint blockade, melanoma, MDSC, monocytic MDSC, granulocytic MDSC

## Abstract

**Introduction:**

Myeloid-derived suppressor cells (MDSC) are a subset of immature myeloid cells that inhibit anti-tumor immunity and contribute to immune therapy resistance. MDSC populations were measured in melanoma patients receiving immune checkpoint inhibitors (ICI).

**Methods:**

Patients with melanoma (n=128) provided blood samples at baseline (BL), and before cycles 2 and 3 (BC2, BC3). Peripheral blood mononuclear cells (PBMC) were analyzed for MDSC (CD33^+^/CD11b^+^/HLA- DR^lo/-^) and MDSC subsets, monocytic (CD14+, M-MDSC), granulocytic (CD15+, PMN-MDSC), and early (CD14-/CD15-, E-MDSC) *via* flow cytometry. Statistical analysis employed unpaired and paired t-tests across and within patient cohorts.

**Results:**

Levels of MDSC as a percentage of PBMC increased during ICI (BL: 9.2 ± 1.0% to BC3: 23.6 ± 1.9%, p<0.0001), and patients who developed progressive disease (PD) had higher baseline MDSC. In patients who had a complete or partial response (CR, PR), total MDSC levels rose dramatically and plateaued (BL: 6.4 ± 1.4%, BC2: 26.2 ± 4.2%, BC3: 27.5 ± 4.4%; p<0.0001), whereas MDSC rose less sharply in PD patients (BL: 11.7 ± 2.1%, BC2: 18.3 ± 3.1%, BC3: 19.0 ± 3.2%; p=0.1952). Subset analysis showed that within the expanding MDSC population, PMN-MDSC and E-MDSC levels decreased, while the proportion of M-MDSC remained constant during ICI. In PD patients, the proportion of PMN-MDSC (as a percentage of total MDSC) decreased (BL: 25.1 ± 4.7%, BC2: 16.1 ± 5.2%, BC3: 8.6 ± 1.8%; p=0.0105), whereas a heretofore under-characterized CD14+/CD15+ double positive MDSC subpopulation increased significantly (BL: 8.7 ± 1.4% to BC3: 26.9 ± 4.9%; p=0.0425).

**Conclusions:**

MDSC levels initially increased significantly in responders. PMN-MDSC decreased and CD14+CD15+ MDSC increased significantly in PD patients. Changes in MDSC levels may have prognostic value in ICI.

## Introduction

Melanoma accounts for the vast majority of skin cancer-associated deaths, and its incidence has risen rapidly over the past 30 years in the United States. It is estimated that over 95,000 new cases of melanoma will be diagnosed in the United States in 2019 (1). Malignant melanoma has been identified as a tumor type with a high mutational burden (2). Melanoma tumors have been shown to harbor a high number of neoantigens which have the potential to induce a robust host immune response. Patients with advanced melanoma were some of the first to receive immune checkpoint inhibitors and promising efficacy was observed (3, 4). This therapeutic approach has revolutionized the treatment of cancer for melanoma and an increasing number of other tumor types (5).

Myeloid-derived suppressor cells (MDSC) are immature myeloid cells with immunosuppressive functions that expand in tumor-bearing hosts in response to tumor-derived factors (6, 7). MDSC frequency is increased in patients with cancer including, renal cell carcinoma, hepatocellular carcinoma (HCC), non-small cell lung carcinoma (NSCLC), glioblastoma, gastrointestinal, breast cancer, prostate cancer and melanoma (8–15). Cytokines, chemokines and metabolites produced by tumor cells lead to aberrant myelopoiesis which results in the generation, expansion and recruitment of MDSC to the tumor site. In humans, MDSC are characterized as CD33^+^, CD11b^+^, and HLA-DR^lo/neg^ (6, 16, 17). Further subsets can be defined, with monocytic MDSC (M-MDSC) being characterized as CD14+/CD15-, and granulocytic MDSC (PMN-MDSC) being identified as

CD14-/CD15+. Early MDSC (e-MDSC) are negative for both CD14 and CD15. MDSC are recruited to the tumor by chemokines such as CXCL3, CXCL5, CXCL12, CCL2, and CCL5 where they mediate their immune suppressive effects (18). MDSC can suppress immune cell function utilizing a variety of mechanisms. This includes the generation of reactive oxygen species (ROS) and nitric oxide (NO), the secretion of IL-10 and TGF-β, as well as the over-expression of arginase and IDO, all of which can lead to the inhibition of T cell function (19). Studies in murine models indicate that disruption of MDSC function can reverse immune tolerance to tumor antigens, stimulate anti-tumor immune responses and improve the efficacy of immune-based therapies such as cancer vaccines and immune checkpoint inhibitors (ICI) (7, 20).

Melanoma has been a major focus for the study of cancer immunotherapy due to the occurrence of spontaneous regression in primary tumors, the presence of tumor-infiltrating lymphocytes (21), and the detection of circulating antigen-specific cytotoxic T cells and antibodies (22). Historically, metastatic melanoma was a disease with an extremely poor prognosis demonstrating a median survival of <1 year (23). The advent of immune checkpoint inhibitors directed against PD-1 (nivolumab) and anti-CTLA-4 (ipilimumab) has increased the overall survival of stage III/IV melanoma patients (24). Follow up studies revealed that only a subset of patients (11-34%) have a clinically significant or lasting response with monotherapy (3, 25, 26), sparking the investigation of combinatorial therapy. Results from the CheckMate-067 study showed enhanced survival in patients receiving ipilimumab plus nivolumab compared to either therapy alone at 5 years. However, a significant number of patients (24%) still did not respond (27).

Since the mechanism behind immunotherapy resistance is not well understood, coupled with the increasing clinical utilization of ICI, there is increasing interest in finding biomarkers that can predict those patients that will respond favorably. Given that the presence of MDSC can attenuate the function of immune cells and potentially inhibit immune-based therapies, strategies that are aimed at depleting or blocking the immune suppressive function of MDSC could be a successful approach to improving the efficacy of checkpoint inhibitors (7, 28). The current study aims to further our understanding of the immunosuppressive milieu in melanoma patients and how it changes with the administration of ICI.

## Materials and Methods

### Study Design and Patient Population

The objective of this prospective study was to elucidate the ongoing changes to systemic MDSC populations in patients with advanced melanoma as they receive ICI. Patients with melanoma who were scheduled to receive ICI therapy were considered eligible for this study. There were no specific restrictions with respect to performance status, organ function, or prior/concurrent modes of therapy, or stage of disease. Samples were drawn at the time of initiation of therapy (baseline, BL), and prior to the beginning of cycles 2 (before cycle 2, BC2) and 3 (before cycle 3, BC3) for a total of 3 blood draws. Eligible patients received pembrolizumab, nivolumab, ipilimumab, or nivolumab in combination with ipilimumab. Treatment intervals were dependent on the therapy received: Pembrolizumab was given every 3 weeks, Nivolumab was given every 4 weeks, and combination ipilimumab and nivolumab was given every 3 weeks. Peripheral blood specimens consisted of approximately 50 mL of blood. Samples were processed as described below.

### Sample Collection and Procurement

Peripheral blood was obtained from patients with melanoma (n=128) following consent to participate in this IRB-approved prospective clinical study (NCI-2020-06536). Peripheral blood mononuclear cells (PBMC) were isolated from peripheral venous blood *via* density gradient centrifugation with Ficoll-Paque, as previously described (28). 1×10^6^ PBMC were processed and analyzed by flow cytometry as described below.

### Assessment of Clinical Response

Patients were followed in the Melanoma Clinic at the OSU James Cancer Hospital and treatment decisions were made by their oncologist. Response to immunotherapy was assessed using Response Evaluation Criteria in Solid Tumors (RECIST) 1.1.

### Flow Cytometry for Myeloid-Derived Suppressor Cells

**Figure 1:**
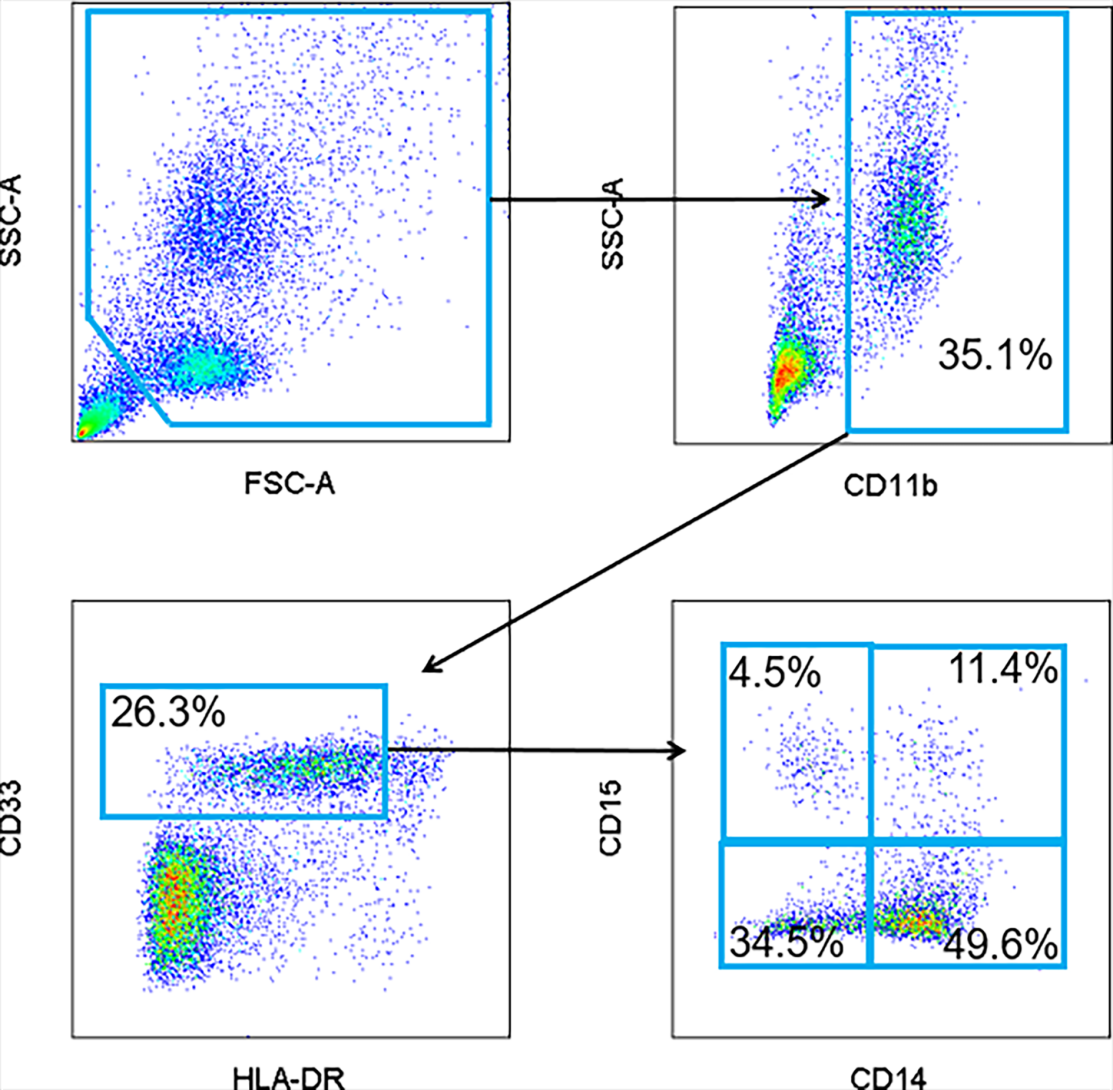
Representative flow cytometry gating strategy for MDSC. Peripheral blood mononuclear cells (PBMC) were isolated from patient peripheral blood samples. Circulating levels of total MDSC (CD11b^+^, CD33^+^, and HLA-DR^lo/-^), granulocytic (CD15^+^), monocytic (CD14^+^) and early (CD14^-^/CD15^-^) MDSC were quantified and visualized by flow cytometry. A population of CD14+/CD15+ double positive cells was also identified.

PBMC were analyzed for the presence of MDSC as previously described (12). Briefly, MDSC were defined as cells positive for CD33, CD11b and with low to no expression of HLA-DR with subsets expressing CD15 or CD14 representing granulocytic and monocytic MDSC, respectively (**Figure 1**). Specific antibodies included CD15-FITC, CD33-APC, HLA-DR-PC7, CD11b-PE (all Beckman Coulter), and CD14-V450 (BD Biosciences). All samples were analyzed on a BD LSR II flow cytometer and the data were subsequently analyzed with FlowJo software. Cells were then categorized into specific subsets: early MDSC (CD14-/CD15-), monocytic MDSC (CD14+/CD15+), granulocytic MDSC (CD14-/CD15+). Notably, there is a sizeable population within the gated cells that were double positive (CD14+/CD15+) that are not traditionally described as MDSC. As such, these cells were analyzed separately.

### Statistical Analysis

Statistical analyses were performed using GraphPad Prism Software. Analyses across and between patient cohorts were performed and p values were generated using unpaired and paired t-tests.

## Results

### Patient Characteristics

A total of 128 patients with melanoma were consented to participate in an IRB-approved prospective clinical registry (OSU-13114). Patients provided blood samples at the initiation of immune checkpoint therapy (before cycle 1 or baseline, BL) and prior to the beginning of cycles 2 (before cycle 2, BC2) and 3 (before cycle 3, BC3). Cycle length was dependent on therapy. Pembrolizumab was given every 3 weeks, nivolumab was every 4 weeks and the combination of ipilimumab and nivolumab was every 3 weeks. Patient characteristics are summarized in **Table 1**. The median age at diagnosis was 60 years and the majority of patients were male (59%, n=75). Initial histology subtypes were as follows: superficial spreading (n=33), nodular (n=28), lentigo maligna (n=13), and acral lentiginous melanoma (n=3). Six patients had an otherwise not specified type of melanoma and forty-eight patients were diagnosed at a late stage and did not have primary pathology slides available for confirmation of histology. Forty percent (n=51) of the patients in this cohort had a known BRAF mutation. The majority of patients had measurable metastatic disease (59%, n=75), and the rest had locally advanced disease (41%, n=53). Most patients had prior surgery (70%). Other prior therapy included chemotherapy (6%), radiation (13%), immunotherapy (31%), and none (16%).

**Table 1:** Demographic data for the 128 patients included in the study are listed. Disease characteristics, prior treatment modalities including immunotherapy and treatment response are included.

	Total N = 128
Age on Dx (years)	
Mean	60
Median	63
Min, Max	8, 96
SD	15.32
Gender, n (%)	
Male	75 (59)
Female	53 (41)
Type of Melanoma, n (%)	
Superficial spreading	33 (26)
Nodular	31 (24)
Lentigo maligna	13 (10)
Acral lentiginous	3 (2)
Other	6 (5)
Unknown	48 (38)
BRAF Mutation, n (%)	
Yes	51 (40)
No	52 (40)
Unknown	25 (20)
Metastasis, n (%)	
Yes	75 (59)
No	53 (41)
Prior Treatment, n (%)	
Surgery	89 (70)
Chemotherapy	8 (6)
Radiation	17 (13)
Immunotherapy	40 (31)
None	21 (16)
Immunotherapy, n (%)	
IFNα	1 (1)
Ipilimumab	4 (3)
Ipilimumab/Nivolumab	11 (9)
Nivolumab	82 (64)
Pembrolizumab	30 (23)
Best Overall Response, n (%)	
Complete Response	31 (24)
Partial Response	18 (15)
Stable Disease	30 (23)
Progressive Disease	32 (27)
Not Evaluable	17 (13)

### Immunotherapy Regimens

**Table 1** summarizes the types of immunotherapy administered. Treatment regimens were chosen by the patient’s primary oncologist. Most patients received single agent nivolumab (n=77). Thirty-five patients received pembrolizumab, eleven patients received the combination regimen of ipilimumab and nivolumab, four received ipilimumab. Agent choice was at the physicians’ discretion.

### Overall Clinical Response in Melanoma Patients Undergoing Immunotherapy for Metastatic Melanoma

**Table 1** also provides a summary of tumor response in the 111 patients who were evaluable for efficacy. Clinical response was evaluated at time of the first restaging scan, most frequently done after 3 cycles of immunotherapy. Overall, 24% of patients had a complete response (CR), 15% had a partial response (PR), 23% had stable disease (SD), and 26% had progressive disease (PD). The remaining 13% of patients could not be assessed, primarily because staging scans had not yet obtained by their oncologist. Notably, of the sixty-eight evaluable patients who received nivolumab, 34% (n=23) experienced a complete response and 12% (n=8) experienced a partial response. Of the thirty-five evaluable patients who received pembrolizumab, 17% (n=6) experienced a complete response, and 29% (n=10) experienced a partial response. With nivolumab, 78% achieved stable disease or better based on RECIST criteria. With pembrolizumab, 74% achieved stable disease or better based on RECIST criteria.

### Levels of Circulating Myeloid-Derived Suppressor Cells at Baseline

**Figure 2:**
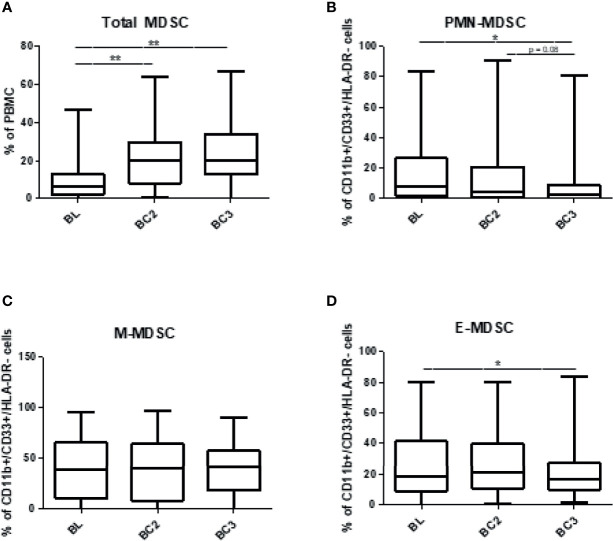
Levels of circulating myeloid-derived suppressor cells over the course of immune checkpoint blockade. Patient PBMCs were isolated at the time of initiation of immune checkpoint therapy (baseline, BL), and prior to the beginning of cycles 2 (before cycle 2, BC2) and 3 (before cycle 3, BC3) and analyzed for total MDSC (CD11b^+^, CD33^+^, HLA-DR^lo/-^) and granulocytic (CD15^+^), monocytic (CD14^+^), or early (CD14-/CD15-) subsets *via* flow cytometry. **(A)** Total MDSC are represented as a percentage of PBMC, and individual subsets represent the proportion of MDSC that fall into each subset: **(B)** PMN-MDSC, **(C)** M-MDSC, and **(D)** E-MDSC Data are shown as box and whisker plots with ranges, *p < 0.05, **p < 0.01.

To determine the changes in the levels of circulating myeloid-derived suppressor cells (MDSC) associated with immunotherapy in melanoma patients, total peripheral blood mononuclear cells (PBMCs) were analyzed prior to cycles 1, 2, and 3 of immunotherapy *via* flow cytometry. The gating strategy is provided in **Figure 1**. MDSC were identified as CD11b^+^, CD33^+^, HLA- DR^lo/neg^ cells. MDSC subsets were further characterized as CD14+ for M-MDSC and CD15+ for PMN‑MDSC. Early MDSC, or E-MDSC, were identified as those that were negative for both CD14 and CD15 (16). A unique population of CD14+/CD15+ double positive cells was also identified and these were included in the MDSC analysis. The characteristics and treatment-induced trajectory of this cell population are discussed at the conclusion of this Results section. At baseline, MDSC comprised 9.2 ± 1.0% of all PBMC. A greater proportion of MDSC were classified as M-MDSC at 39.5 ± 3.2% compared to 16.4 ± 2.2% for PMN-MDSC **(****Figure 2****)**, and the remaining MDSC were 27.7 ± 2.4% E‑MDSC. The double positive population comprised the remainder of the gated CD11b^+^, CD33^+^, HLA- DR^lo/neg^ cells (13.9 ± 1.7%).

**Figure 3:**
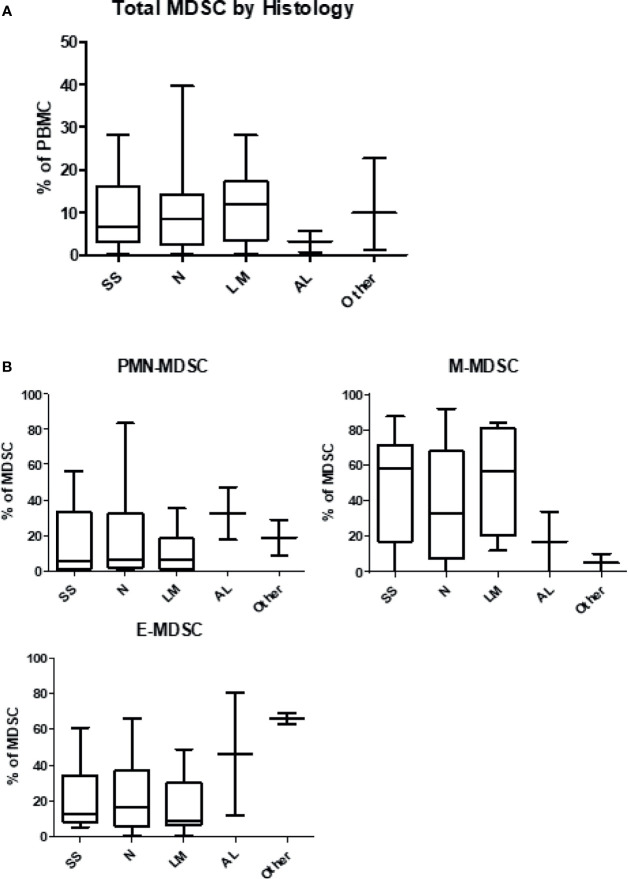
Levels of myeloid-derived suppressor cells according to histologic type of melanoma. Patient PBMCs were isolated from blood draws at the time of initiation of immune checkpoint therapy, and analyzed for MDSC levels and individual subsets. **(A)** Circulating levels of total MDSC according to histologic type of melanoma. **(B)** Circulating levels of granulocytic (PMN-MDSC), monocytic (M-MDSC) and early (E-MDSC) MDSC by type of melanoma. SS, Superficial Spreading; N, Nodular; LM, Lentigo Maligna; AL, Acral Lentiginous.

Patient MDSC levels were then evaluated according to melanoma histology type (**Figures 3A, B**). Notably, the levels of total MDSC were lowest at baseline in patients with acral lentiginous melanoma at 3.3%, while other subtypes had approximately 10% MDSC at baseline (superficial spreading: 9.8 ± 1.9%; nodular: 10.7 ± 2.4%; lentigo maligna: 11.6 ± 2.8%; other: 11.4 ± 6.3%). Patients with lentigo maligna melanoma had the lowest levels of PMN-MDSC at 10.1 ± 3.7% of the total population but the highest levels of M-MDSC at 51.6 ± 8.8%, whereas acral lentiginous patients had the highest levels of PMN-MDSC at 32.4 ± 14.8% but the lowest levels of M-MDSC at 16.9 ± 4.6%. Patients with acral lentiginous melanoma had the highest levels of E-MDSC among the 4 skin melanoma types, and patients with extra-dermal melanomas (other) also had high levels of E-MDSC. The differences in MDSC subset proportions between different melanoma subtypes were consistent but did not reach statistical significance. Individual dot plots of this data are visualized in **Supplemental Figures 1, 2**.

### Levels of Circulating Myeloid-Derived Suppressor Cells Following Immune Checkpoint Blockade

Overall MDSC numbers were calculated as a percentage of PBMC and the proportions of each MDSC subtype were calculated from all CD11b^+^, CD33^+^, HLA- DR^lo/neg^ cells. When assessing the individual MDSC subsets, several trends were identified. The frequency of total MDSC in PBMC significantly increased after two cycles of immunotherapy in the total population of patients (BL: 9.2 ± 1.0% to BC3 23.6 ± 1.9%; p<0.0001). An analysis of the subset composition of MDSC showed that the PMN-MDSC population responded differently after immunotherapy with the proportion of PMN-MDSC decreasing significantly over 3 cycles (BL: 16.4 ± 2.2% to BC3: 9.1 ± 2.0%; p=0.0196). Similarly, E-MDSC also decreased after the initiation of immunotherapy (BL: 27.7 ± 2.5% to BC3: 21.7 ± 2.3%; p=0.0392). Over this time span, levels of M-MDSC did not display such a striking decrease; their levels remained unchanged throughout the course of immunotherapy (BL: 39.5 ± 3.2% to BC3: 38.5 ± 3.2%) (**Figure 2**). It is important to note that these trends in MDSC subset composition took place in the context of an overall increase in MDSC numbers over three cycles of therapy.

### Myeloid-Derived Suppressor Cells and Response to Immunotherapy

**Figure 4:**
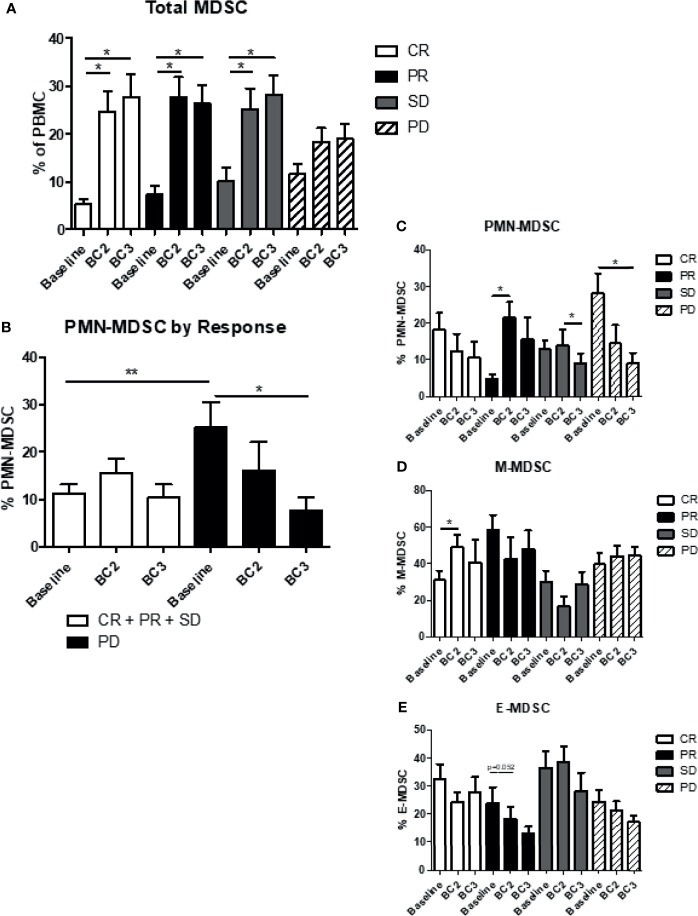
Myeloid-derived suppressor cells and response to immunotherapy. Patient MDSC levels were evaluated according to immunotherapy response, namely clinical benefit (CR + PR + SD) or progressive disease (PD). **(A)** Levels of total MDSC grouped by patient response to immunotherapy. **(B)** Levels of granulocytic (PMN-MDSC) in the two response categories are shown. Levels of **(C)** PMN-MDSC, **(D)** monocytic MDSC (M-MDSC) and **(E)** early MDSC (E-MDSC). Data are shown as mean ± SE, *p < 0.05, **p < 0.01. CR, complete response; PR, partial response; SD, stable disease; and PD, progressive disease.

The correlation between levels of MDSC and the patient response to immunotherapy was analyzed. As noted above, the overall frequency of total MDSC in the blood (CD11b^+^, CD33^+^, HLA-DR^lo/-^) increased significantly from baseline after just one cycle of immunotherapy in all patients. This effect was maintained after two cycles of immunotherapy in patients with clinical benefit, including those with a complete response (CR; BL: 5.3 ± 1.1%, BC2: 24.7 ± 4.2%, BC3: 27.7 ± 4.7%; p=0.0002), partial response (PR; BL: 7.5 ± 1.6%, BC2: 27.7 ± 4.2%, BC3: 26.3 ± 3.9%; p=0.0024), and stable disease (SD; BL: 10.1 ± 2.9%, BC2: 25.3 ± 4.3%, BC3: 28.3 ± 4.0%; p=0.0138). Total MDSC levels also increased in patients who exhibited overall progressive disease, although to a lesser and non-significant extent (PD; BL: 11.7 ± 2.1%, BC2: 18.3 ± 3.1%, BC3: 19.0 ± 3.2%; p=0.1952) (**Figure 4A**).

Changes in circulating levels of CD15^+^ PMN-MDSC and CD14^+^ M-MDSC varied depending on the patient response to immunotherapy. Patients who showed clinical benefit had a significantly lower amount of PMN-MDSC at baseline compared to those with PD (CR+PR+SD: 10.7 ± 1.6% *vs* PD: 26.2 ± 5.4% p=0.0147). PMN-MDSC increased marginally after one cycle of immunotherapy in the clinical benefit population and then decreased to baseline levels after the second cycle of immunotherapy (BL: 10.7 ± 1.6%, BC2: 15.3 ± 2.2%, BC3: 10.2 ± 2.4%), whereas PMN-MDSC levels declined at BC2 and BC3 for PD patients (BL: 25.1 ± 4.7%, BC2: 16.1 ± 5.2%, BC3: 8.6 ± 1.8%; p=0.0105) (**Figure 4B**). Indeed, when comparing the four categories of response, the most significant decrease in PMN-MDSC after two cycles of immunotherapy was seen in patients with progressive disease (p=0.0105) (**Figure 4C**). M-MDSC levels remained largely stable through the course of immunotherapy regardless of response category. There was, however, a significant increase in M-MDSC levels from 30.4 ± 4.8% at baseline to 48.8 ± 8.2% (p=0.0392) after one cycle of immunotherapy in complete responders, but this effect dissipated after a second cycle of immunotherapy (**Figure 4D**). Early MDSC trended downward during therapy across all groups but this trend did not reach statistical significance (**Figure 4E**).

### The Effects of Prior Immune Therapy on Circulating MDSC Levels

**Figure 5:**
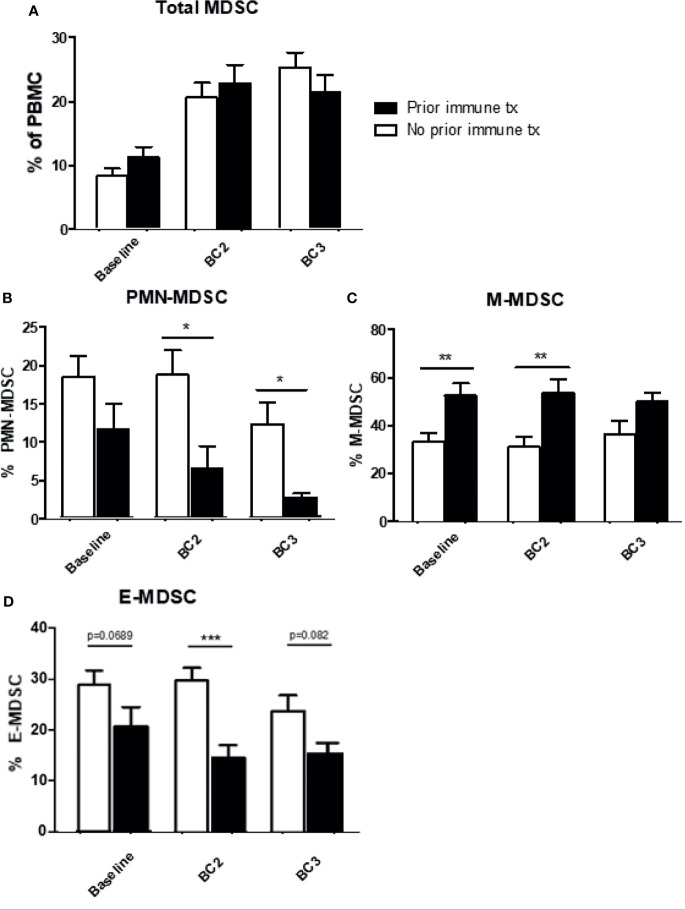
Effects of prior immune system modulation on circulating MDSC levels. Levels of MDSC according to the receipt of prior immunotherapy. Levels of **(A)** total **(B)** granulocytic MDSC (PMN-MDSC), **(C)** monocytic MDSC (M-MDSC), and **(D)** early MDSC (E-MDSC) are shown. Data are shown as mean ± SE, *p < 0.05, **p < 0.01, ***p < 0.001.

The effect of prior immunotherapy on MDSC levels was evaluated. Most of these patients (n=80) had received either IFN-α therapy (26 of 40) or cytokine therapy (14 of 40) and none had received prior checkpoint inhibitors. Patients were divided into two groups: those who received prior immunotherapy and those who did not, and their MDSC levels were analyzed prior to cycles 1, 2, and 3 of current immune checkpoint inhibition. The frequency of total MDSC (CD11b^+^, CD33^+^, HLA-DR^lo/neg^) increased across draws 1-3, regardless of whether patients had received prior immunotherapy or not (**Figure 5A**). Those patients who had previously received immunotherapy exhibited lower levels of PMN-MDSC and higher levels of M-MDSC at baseline and after each cycle of therapy. Following initiation of checkpoint inhibition, PMN-MDSC and M-MDSC had very different responses in these two groups (**Figures 5B, C**). Although circulating levels of PMN-MDSC decreased in both groups of patients, this decrease was significantly pronounced in patients with prior immunotherapy (No prior: BL: 18.5 ± 4.2% to BC3 12.1 ± 2.2% *vs.* prior: BL: 12.3 ± 2.6% to BC3 2.3 ± 0.6%; p=0.0174). M-MDSC levels, while elevated in patients with a history of immunotherapy as compared to those with no history (BL: 53.2 ± 3.6% *vs.* 36.1 ± 2.2%, p=0.0045), did not change significantly over the course of/itali treatment (**Figure 5C**). Early MDSC were higher at all time points in prior immune therapy patients, but levels remained relatively stable in both groups (**Figure 5D**).

### Levels of Circulating MDSC Based on the Type of Immunotherapy

**Figure 6:**
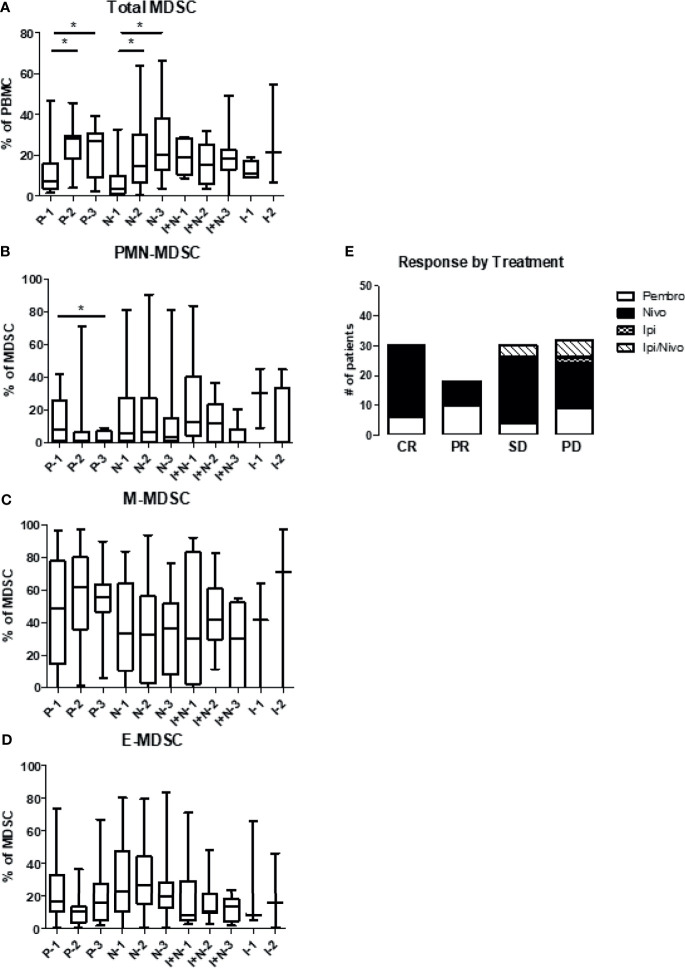
Levels of circulating MDSC based on the type of immunotherapy. Patient PBMCs were evaluated for MDSC levels and analyzed according to type of checkpoint inhibitor therapy received. Patients received either pembrolizumab (Pembro), nivolumab (Nivo), ipilimumab/nivolumab (Ipi/Nivo), or ipilimumab alone (Ipi). Levels of **(A)** total MDSC **(B)** granulocytic MDSC (PMN-MDSC), **(C)** monocytic MDSC (M-MDSC) and **(D)** early MDSC (E-MDSC) are shown. **(E)** Clinical response is stratified based on type of immunotherapy given. Data are shown as box and whisker plots with ranges, *p < 0.05.

**Figures 6A–C** summarizes the levels of total MDSC, PMN-MDSC, and M-MDSC by type of immune therapy received. Patients were grouped as having received pembrolizumab, nivolumab, ipilimumab and nivolumab in combination, or ipilimumab alone. As noted previously, levels of total circulating MDSC within the PBMC compartment increased over all cycles in all treatment groups except the combination patients. Patients receiving pembrolizumab or nivolumab displayed significant increases in levels of total MDSC after one cycle of immunotherapy. However, PMN-MDSC levels significantly decreased (12.4 ± 2.7% to 2.8 ± 0.8%) from baseline after 2 cycles of pembrolizumab. Patients receiving nivolumab also saw decreasing levels of PMN-MDSC from baseline after two cycles but to a lesser extent (17.8 ± 2.2% to 12.7 ± 3.1%) (**Figure 6B**). M-MDSC levels remained relatively constant across draws 1-3 in patients receiving pembrolizumab, nivolumab or ipilimumab/nivolumab in combination. (**Figure 6C**). Finally, E-MDSC seemed to decrease with all checkpoint inhibitors with the most significant drop seen in the nivolumab group (**Figure 6D**). We then correlated treatment responses with the type of immunotherapy regimen given (**Figure 6E**). Individual dot plots were visualized in **Supplemental Figure 3**.

### Characterization of a CD14+/CD15+ MDSC Subpopulation

**Figure 7:**
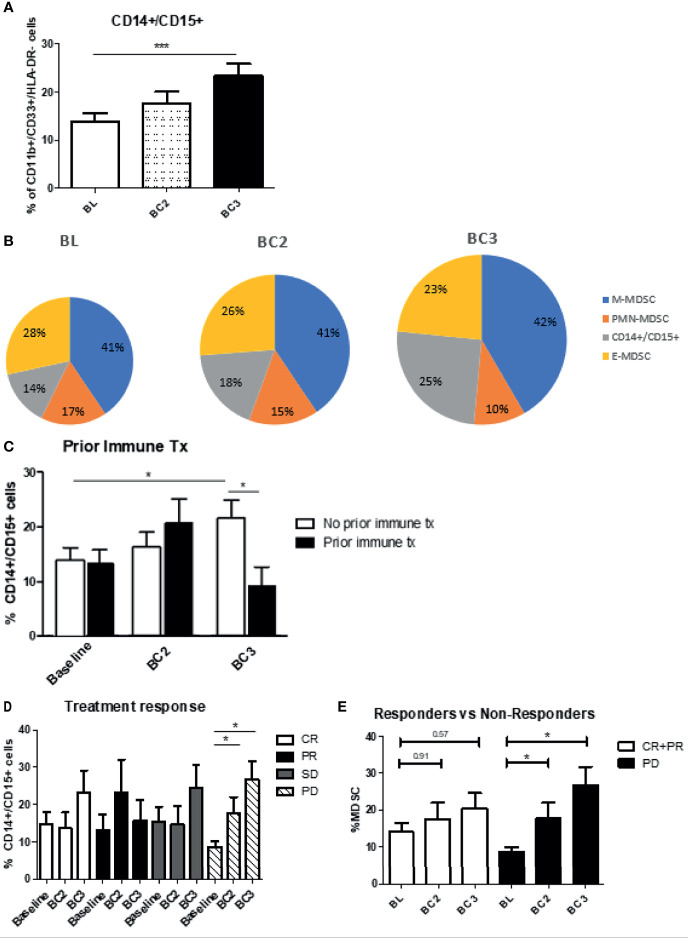
Levels of CD14/CD15 double positive cells. **(A)** Patient PBMCs were evaluated for CD14/CD15 double positive cells and analyzed over the course of immune checkpoint blockade. **(B)** Changes to the proportions of the different MDSC subsets along with these double positive cells are shown over the course of immune checkpoint blockade, with size of pie chart corresponding to increasing MDSC percentage. Levels of the double positive cells were analyzed with regards to **(C)** prior immune therapy, **(D)** clinical response, **(E)** responders *vs* non-responders. Data are shown as mean ± SE, *p < 0.05, ***p < 0.001.

While analyzing the different MDSC subsets, a subpopulation of cells that were positive for both CD14+ and CD15+ was identified. At baseline, these cells represented a small proportion of MDSC, but this proportion significantly increased after checkpoint inhibitor therapy (BL 13.9 ± 1.7% *vs* BC3: 23.3 ± 2.6%; p<0.0007) (**Figure 7A**). The proportions of the different CD11b+/CD33+/HLA-DR^lo/neg^ subsets are shown over the course of checkpoint inhibition with the size of the pie chart indicating the overall levels of MDSC relative to baseline (**Figure 7B**). In contrast to the overall trend, in patients who have had previous immune therapy, double positive cells decreased instead of increasing after two cycles of checkpoint inhibition (No prior: 14.2 ± 2.2% at BL to 21.8 ± 3.3% at BC3, prior: 13.3 ± 2.6% at BL to 9.2 ± 3.6% at BC3; p=0.0251) (**Figure 7C**). When grouping by clinical response, we note that progressive patients start with the lowest percentage of these double positive cells, but increase to have the highest percentage by cycle 3 **(****Figure 7D****)**. When we compared responders versus non-responders, patients with either complete or partial response had a non-significant increase (BL: 14.09 ± 2.5% to BL3: 20.38 ± 4.3%; p=0.57), whereas patients with progressive disease had a significant increase (BL: 8.7 ± 1.4% to BC3: 26.9 ± 4.9%; p=0.0425) (**Figure 7E**).

## Discussion

The effect of immune checkpoint inhibitor treatment on MDSC populations in patients with advanced melanoma was evaluated. In this patient population roughly 10% of PBMC were phenotyped as MDSC at baseline. In addition to these well-described MDSC subtypes, a fourth group of cells that were double positive for CD14 and CD15 was identified. Following the initiation of immune checkpoint blockade, overall levels of circulating MDSC increased in melanoma patients. Responders to immunotherapy had MDSC levels that initially rose dramatically and then plateaued. MDSC levels rose to a lesser extent in patients who progressed on immunotherapy. Also, it was observed that patients with progressive disease had the highest baseline level of MDSCs, as well as the smallest increase in MDSC numbers following checkpoint inhibition. When evaluating the proportions of the different MDSC subsets, it was found that M-MDSC were the most prevalent subtype at 41%, followed by E-MDSC at 28%, and PMN-MDSC at 17%. Notably, the various MDSC subsets behaved differently after immune checkpoint blockade. The proportion of PMN-MDSC and E-MDSC levels decreased, while the proportion of M-MDSC remained largely constant. At the same time, the proportion of CD14+/CD15 double positive cells increased significantly. In PD patients, the proportion of PMN-MDSC decreased significantly, whereas a heretofore under-characterized CD14+/CD15+ double positive MDSC subpopulation increased significantly from 8.7 ± 1.4% to BC3: 26.9 ± 4.9%; p=0.0425). There were differences in MDSC levels in relation to the histologic subtype of the primary cancer, with the lowest baseline levels being seen in acral lentiginous primaries. These findings further define the characteristics and behavior of this inhibitory immune cell population and provide justification for future exploration of their role in modulating the response to immune therapies.

There are multiple mechanisms by which MDSC exert their immunosuppressive effects, and the recruitment of MDSC into tumors represents a distinct mechanism for suppression of the anti-tumor immune response (29). In this study, levels of circulating MDSC in patients with advanced melanoma increased from baseline over the course of three cycles of immunotherapy. The most significant increase was seen immediately after the first dose of ICI, and this effect was sustained over the 3-month time course of the study. The mechanisms underlying this upward MDSC trajectory are not clear. However, it should be noted that systemic levels of immune suppressor cells may not mirror those within the tumor (30). It is also possible that ICI-initiated perturbation of the local tumor microenvironment might lead to the release of cytokines with the ability to induce the generation of MDSC (31). These events might be viewed as evidence of an immunologic “arms race” between malignant cells and activated T cells (32).

Immune checkpoint inhibitors have provided durable responses in patients with advanced-stage melanoma, as well as a variety of other solid tumors. However, a subset of patients become resistant or do not initially respond to immunotherapy (33). One possible contributor to resistance are immune suppressive cells such as MDSC. MDSC have been previously characterized as potential mediators of resistance to ICI (34–36). The different MDSC subsets, granulocytic and monocytic, have been studied and seem to have differential effects in their ability to suppress the T cell compartment. Levels of granulocytic MDSC (PMN-MDSC) have been shown to consistently repress T cell responses and correlate with overall survival (15). Studies have shown that higher levels of PMN-MDSC significantly increases the risk of disease progression, which suggests the potential positive effects of targeting MDSC in the context of checkpoint blockade (14). The actual proportion of PMN-MDSC within the expanding MDSC population significantly decreased in this cohort of melanoma patients. The initiation of immune checkpoint blockade resulted in the preferential depletion of PMN-MDSC, and the most significant decrease in PMN-MDSC after two cycles of immunotherapy was seen in patients with progressive disease. Pembrolizumab and nivolumab showed similar effects on MDSC, but with pembrolizumab having a more pronounced effect on PMN-MDSC. In addition, melanoma patients who went on to develop progressive disease following ICI had higher levels of PMN-MDSC at baseline as compared to patients who exhibited clinical benefit. The present data suggests that ICI may have a greater modulatory effect on the PMN-MDSC population, which may be independent of the patient’s clinical response.

Efforts have been made to find effective biomarkers to help better characterize or predict clinical response to immunotherapy. Martens et al. found that lower levels of LDH and lower levels of CD14+ MDSC correlated with better clinical response to ipilimumab therapy (37). In addition, this group found that the occurrence of adverse events did not correlate with either baseline biomarker signatures or the clinical benefit of ipilimumab. Meyer et al. found that following ipilimumab treatment, MDSC levels remained stable. They reported that responders had overall lower levels of CD14+ MDSC compared to non-responders (28). Similarly, Pico de Coana et al. showed that high levels of classical monocytes and low levels of M-MDSC correlated with improved response rates and overall survival in patients receiving pembrolizumab or nivolumab (38). They also reported that PD-L1 expression in MDSC was significantly increased in patients with shorter progression-free survival and correlated inversely with overall survival. Weber et al. found that high numbers of MDSC were associated with poor survival in patients receiving nivolumab after progressing on ipilimumab (39). The present study showed that patients with progressive disease indeed had higher baseline levels of MDSC. Responders to immunotherapy had MDSC levels that rose dramatically after the start of PD-1 blockade and then plateaued. MDSC levels rose to a lesser extent in patients who progressed on immunotherapy. Notably, levels of CD14+ M-MDSC did not change significantly over the course of therapy. The lack of predictive value for M-MDSC in the present study might reflect differences in gating technique or the focus on early treatment time points.

A population of MDSC positive for both CD14 and CD15 was identified and these so-called “double positive” MDSC exhibited the greatest increase in patients who developed progressive disease following the initiation of ICI. Nishimoto et al. showed in a rheumatoid arthritis model that CD14/CD15 double positive cells were seen transiently in the differentiation process from stem cells to monocytes (40). Veglia et al. recently analyzed CD14 expression in murine and human neutrophils, and showed that CD15+ neutrophils with the highest expression of CD14 had significant upregulation of MDSC effector pathways, including IL-6, NO and ROS production, suggesting an “activated” MDSC phenotype (41). Although we observe a similar downward trend in PMN-MDSC in both complete responders and those who have progressive disease, we see a significant increase in the double positive population in non-responders to therapy, compared to those who do show clinical response. This shift in proportions within the MDSC population toward a more potent, activated immunosuppressive phenotype may help explain the difference in clinical response to therapy. These findings suggest that the double positive population could serve as a biomarker for progression of disease on CPI as well as potential target for therapy to overcome resistance. Further study of the immune suppressive capabilities of this cell population in melanoma patients is underway.

The histologic type of melanoma appears to play a role in the quantity and composition of the MDSC population. In the present study, the majority of patients had melanomas of either the superficial spreading or nodular type, with fewer patients presenting with the acral lentiginous and lentigo maligna types. The baseline level of total MDSC were fairly equal across all melanoma histology types, except for the acral lentiginous melanomas, which had notably less total MDSC. Observations of an impact of the melanoma primary histology on MDSC patterns has not been previously described and may reflect biologic differences in tumor behavior at the molecular level. Notably, there appears to be distinct variability in the proportion of PMN‐MDSC to M-MDSC in tumors arising from different tissues, which indicates that the biology of the primary tumor can have a distinct effect on MDSC generation (42).

In summary, melanoma patients have high levels of MDSC prior to therapy, and levels increase over the treatment course when checkpoint inhibitors are employed. Although total MDSC numbers increased over the course of ICI with PD-1 blocking antibodies, the proportion of PMN-MDSC within the overall population decreased significantly. At the same time, the proportion of double positive (CD14+/CD15+) cells increased over the course of therapy, most significantly in patients who progressed on immunotherapy. Future investigations will identify the tumor factors that govern these changes in MDSC levels.

## Data Availability Statement

The raw data supporting the conclusions of this article will be made available by the authors, without undue reservation.

## Ethics Statement

The studies involving human participants were reviewed and approved by The Ohio State University Institutional Review Board. The patients/participants provided their written informed consent to participate in this study.

## Author Contributions

Authors SS and BB contributed equally to the manuscript. SS, BB, and WC were involved in study conception and design. Experiments and collection and assembly of data were conducted by SS, BB, HSa, GL, LG, DA, EN, MD, AS, MD, LS-K, and AC. All authors were involved in data analysis and interpretation. Administrative support was provided by SS, and WC. LY provided statistical support and analysis. HH, HSh, KK, and WC were involved in the provision of patient samples. SS, BB, LS-K, RW, and WC were involved in manuscript writing. Final approval of the manuscript was provided by all authors.

## Funding

This work was supported by the National Institutes of Health Grants T32AI 106704-01A1 (SS) and UM1 CA186712 (WC).

## Conflict of Interest

The authors declare that the research was conducted in the absence of any commercial or financial relationships that could be construed as a potential conflict of interest.

## Publisher’s Note

All claims expressed in this article are solely those of the authors and do not necessarily represent those of their affiliated organizations, or those of the publisher, the editors and the reviewers. Any product that may be evaluated in this article, or claim that may be made by its manufacturer, is not guaranteed or endorsed by the publisher.
