# Supplementary figures and images for: Effect of Immune Checkpoint Blockade on Myeloid-Derived Suppressor Cell Populations in Patients With Melanoma
Source: Front Immunol. 2021 Oct 12;12:740890. doi: 10.3389/fimmu.2021.740890 (PMC8547308; doi:10.3389/fimmu.2021.740890)

Figure S1

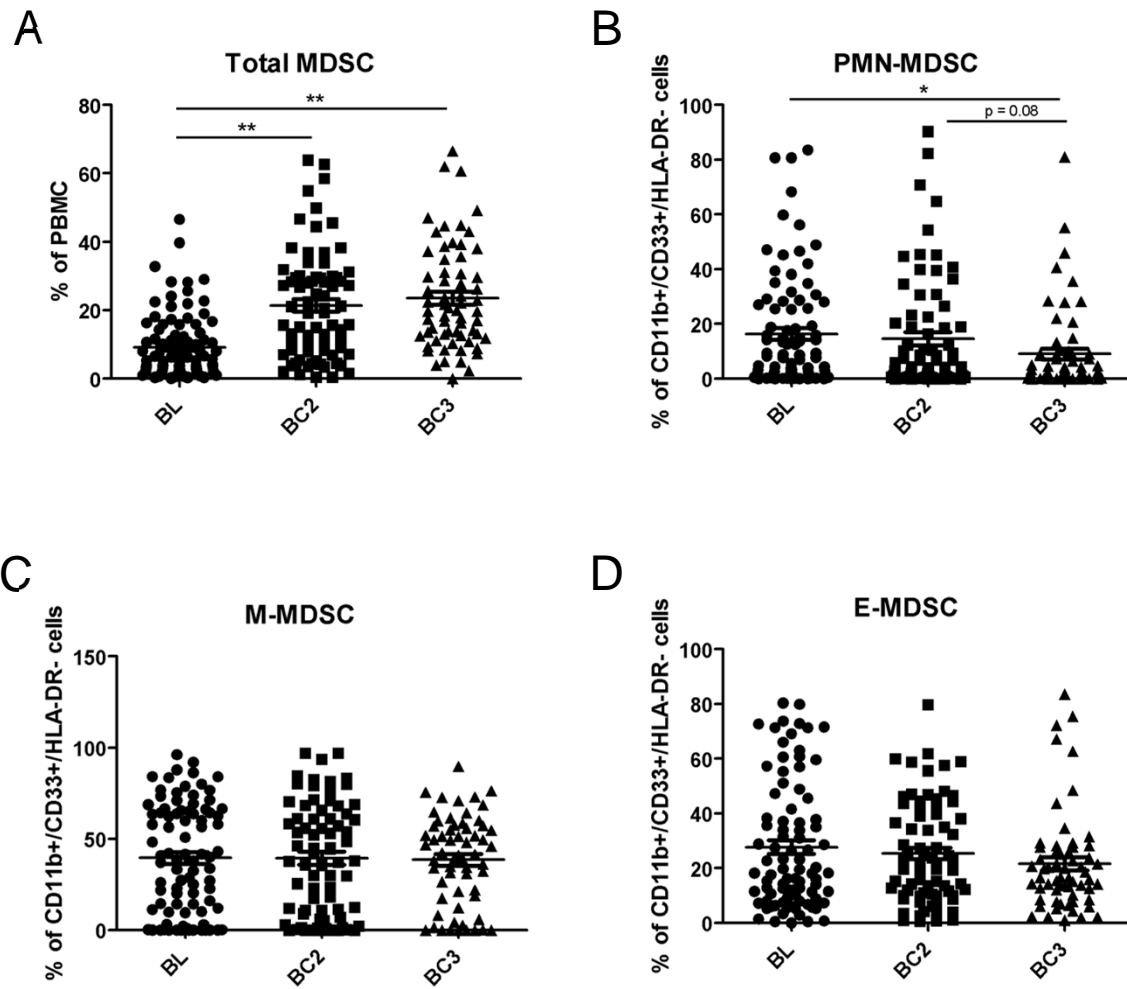

Figure S2

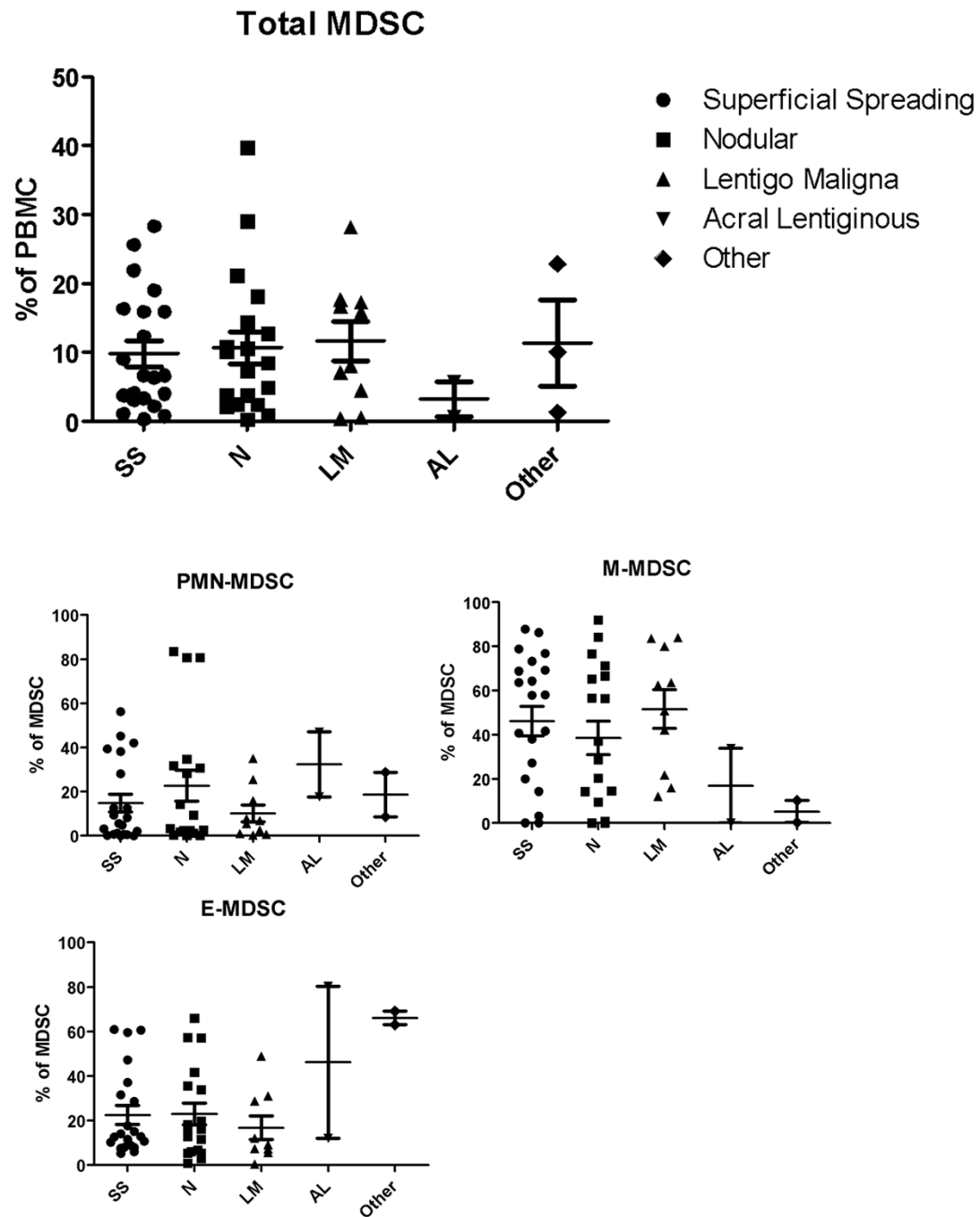

Figure S3

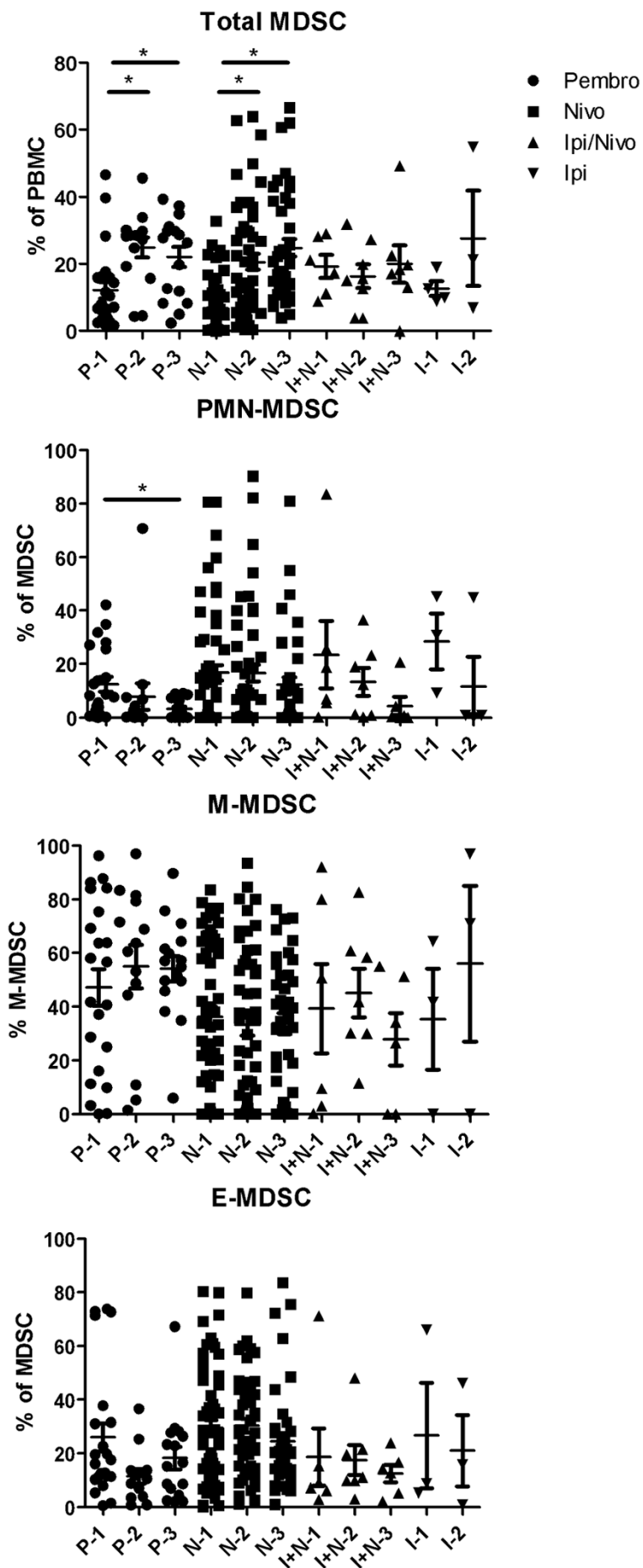

Supplement: Supplementary file 1 [file DataSheet_1.pdf]
